# The Congress on Tuberculosis

**Published:** 1902-11-01

**Authors:** 


### The Hospital.

A Journal of

The Medical Sciences and Hospital Administration.

Vol. XXXIII.—No. 840.] Edited by Sir Henry Burdett, K.C.B., and Solomon C. Smith, M.D., M.R.C.P. [November 1, 1902.

### THE CONGRESS ON TUBERCULOSIS.

The International Congress on Tuberculosis, which was opened at Berlin on the 23rd instant, appears to have excited but a moderate degree of interest and attention in this country, and to have been but scantily attended by Englishmen. Drs. Heron, Hillier, Theodore Williams and Raw are the only countrymen whose names we have so far seen noticed in the reports; and two, if not three, of these gentlemen were present in an official capacity rather than as a result of any spontaneous interest in the proceedings. It would hardly have been possible, indeed, for the organisers of the Congress to have pitched upon a time of meeting less convenient for English practitioners; a time when the medical sessions, alike in London and in provincial cities, have just commenced, and when men in teaching positions are fully engaged in the discharge of their proper duties. It is not impossible that some dissatisfaction with the arrangements may have been felt in this country on other grounds; but, however this may be, it is at least certain that many Englishmen of repute have been conspicuous by their absence, and that the Congress, whatever degree of success may wait upon its deliberations, has derived comparatively little assistance from this country.

The principal feature of the meeting, in so far as its proceedings have as yet been published, and in addition to the reiteration by Professor Koch, seemingly on no basis of further experiment, of his opinion concerning the non-identity of human and bovine tuberculosis, appears to have been a collective visit of the delegates to a vast so-called sanatorium, or house of recovery, at Beelitz, an hour's journey from Berlin, in the Brandenburg pine forest. This is described as an almost palatial establishment, and as an "Imperial Institution," which has arisen out of the system of compulsory assurance for aged and invalid members of the working classes. Under this system, a working man or woman who becomes a permanent invalid obtains a State pension; but, according to the *Times* correspondent, it has been found economical to establish throughout the Empire great sanatoria where "apparently permanent" invalids may be treated for thirteen or fourteen weeks with every advantage of comfortable quarters, pure air, good food, and luxurious baths, in order to give them a chance of recovering their health and of resuming their occupations. The sanatoria at Beelitz, we are further told, are equally magnificent in their architecture, their situation, and their sanitary and engineering appliances. They are evidently calculated to excite the envy of "progressive" municipalities on this side of the Channel, and to produce at least some endeavours to imitate them, with the not wholly unimportant difference that the funds would have to be abstracted from the pockets of the ratepayers, instead of being furnished by a "compulsory assurance" which would require from the "working man" some slight curtailment of his present facilities for the purchase of beer. The "sanatoria" in question seem at first sight to suggest that, if people supposed to be "permanent invalids" do really, in any considerable proportion, become restored to their occupations by living for a few weeks in such an establishment, the arts of diagnosis and prognosis must be less advanced in Germany than supposed.

Such sanatoria, morever, however interesting they may be as objects of study, or however beneficial to those for whom they are intended, do not appear to have much practical bearing upon the subject of tuberculosis, which would comparatively seldom become a source of apparently permanent disability to aged workpeople. There is a well-known scene in "Esmond," in which the chief character says, "Had the gracious lover been happy, he had not passed his time in sighing," and it is almost a fair inference that, if the members of the Congress had been in a position to impart any very important information with regard to the avowed object of their coming together, they would not have devoted the whole of their second day of meeting to a long country journey for the inspection of convalescent hospitals. In truth, we are disposed to think that the probable utility of these international congresses has been greatly exaggerated, and that the prospect of curing tuberculosis by "open-air" or any other treatment is far smaller than many would be inclined to admit. We think it was Dr. Theodore Williams who lately pointed out that it was far more prudent to speak of the "arrest" than of the "cure" of phthisis; and this, which is true even of comparatively wealthy patients, must be still more applicable to those who, when the arrest has been accomplished, are compelled by circumstances to return to crowded homes, unhealthy occupations, or other unfavourable conditions of living. We cannot but think that our chief hope for the future in regard to tuberculosis, more especially as it attacks the wage-earner or the comparatively poor, will be found to rest upon the steady advancement of general sanitation and upon the improvement of industrial conditions, rather than upon attempts, however benevolent or well-intended, to restore health to the actual victims of the disease. Even as regards the attainment of the latter object, we should place more reliance upon the hospital and the clinical physician than upon the orator at congresses, however eloquent and accomplished he might be.

